# Use of electronic health record data to identify skin and soft tissue infections in primary care settings: a validation study

**DOI:** 10.1186/1471-2334-13-171

**Published:** 2013-04-10

**Authors:** Pamela J Levine, Miriam R Elman, Ravina Kullar, John M Townes, David T Bearden, Rowena Vilches-Tran, Ian McClellan, Jessina C McGregor

**Affiliations:** 1 Department of Pharmacy Practice, College of Pharmacy, Oregon State University/Oregon Health & Science University, 3303 SW Bond Avenue CH12C, Portland, Oregon, 97239, USA; 2 Division of Infectious Diseases, Department of Medicine, School of Medicine, Oregon Health & Science University, 3181 SW Sam Jackson Park Road L457, Portland, Oregon, 97239, USA

**Keywords:** Abscess, Primary care, Skin infection, Methodologies, Positive predictive value

## Abstract

**Background:**

Epidemiologic studies of skin and soft tissue infections (SSTIs) depend upon accurate case identification. Our objective was to evaluate the positive predictive value (PPV) of electronic medical record data for identification of SSTIs in a primary care setting.

**Methods:**

A validation study was conducted among primary care outpatients in an academic healthcare system. Encounters during four non-consecutive months in 2010 were included if any of the following were present in the electronic health record: International Classification of Diseases, Ninth Revision (ICD-9) code for an SSTI, Current Procedural Terminology (CPT) code for incision and drainage, or a positive wound culture. Detailed chart review was performed to establish presence and type of SSTI. PPVs and 95% confidence intervals (CI) were calculated among all encounters, initial encounters, and cellulitis/abscess cases.

**Results:**

Of the 731 encounters included, 514 (70.3%) were initial encounters and 448 (61.3%) were cellulitis/abscess cases. When the presence of an ICD-9 code, CPT code, or positive culture was used to identify SSTIs, 617 encounters were true positives, yielding a PPV of 84.4% [95% CI: 81.8–87.0%]. The PPV for using ICD-9 codes alone to identify SSTIs was 90.7% [95 % CI: 88.5–92.9%]. For encounters with cellulitis/abscess codes, the PPV was 91.5% [95% CI: 88.9–94.1%].

**Conclusions:**

ICD-9 codes may be used to retrospectively identify SSTIs with a high PPV. Broadening SSTI case identification with microbiology data and CPT codes attenuates the PPV. Further work is needed to estimate the sensitivity of this method.

## Background

In recent years, ICD-9 diagnosis codes have been more commonly used for retrospective identification and classification of SSTIs from administrative and electronic health records (EHR).[1-6] Although this is a simple and straightforward method for case identification, these data are not collected for research purposes and may be subject to misclassification. While ICD-9 code validity has been assessed for other infections and diseases, there have been no reported evaluations of their use in the retrospective identification of SSTIs. The inherently subjective nature of various medical diagnoses combined with extrinsic factors such as human error or delays in data entry may affect the validity of these codes. The variability in ICD-9 code validity for identifying specific diagnoses depends upon the disease studied and clinical setting, as well as the specific algorithm used for case identification (i.e., which specific ICD-9 codes were used and if other clinical data were included).[7-10] Further, in scenarios where there is no definitive gold standard, the definition of ‘true’ disease may impact positive predictive value (PPV).[10] Consequently, it is crucial to assess the validity of using diagnosis codes to detect specific cases of interest, such as SSTI infections.

Since valid methods of case ascertainment are critical to minimize the effects of misclassification in epidemiologic and outcomes studies of SSTIs, our primary objectives in this study were to (1) estimate the PPV of ICD-9 diagnosis codes for the retrospective identification of SSTIs in an outpatient primary care setting and (2) determine whether modifying the SSTI identification algorithm to include additional diagnostic indicators (i.e., wound culture and incision and drainage) would improve the precision of prediction results.

## Methods

A validation study was conducted among ambulatory primary care patients at Oregon Health & Science University (OHSU). OHSU is a large academic healthcare system that includes two hospitals and numerous outpatient clinics throughout the greater Portland, Oregon metropolitan area; the OHSU healthcare system has over 750,000 patient encounters annually. The EpicCare EHR system (Epic Systems) is used for both inpatient and outpatient encounters throughout this system. For this study, encounters occurring in January, April, July or October 2010 in outpatient, non-specialty clinics of the Family Medicine, Internal Medicine and Pediatrics departments were eligible for inclusion; these months were selected to be representative of the calendar year and minimize seasonal or secular variations in the data. Patient encounters were included if any of the following criteria were present in the EHR: an SSTI ICD-9 diagnosis code [erysipelas: 035; carbuncle and furuncle: 680.x; cellulitis and abscess: 681.0, 681.00, 681.01, 681.10, 681.9, 682.x; acute lymphadenitis: 683.x; impetigo: 684.x; other local infections of skin and subcutaneous tissue: 686.x; other specified diseases of hair and hair follicles: 704.8], Current Procedural Terminology (CPT) code for incision and drainage (IND) [10060/1, 10080/1, 10120/1, 10140, 10160, 10180], or a positive wound or tissue microbiology culture. Encounters with missing electronic data were excluded. This study was approved by the Oregon Health & Science University institutional review board.

### Patient identification and data collection

Data were electronically abstracted from the institution’s research data warehouse (RDW), a data repository that stores clinical, laboratory, and administrative data from the electronic medical record data systems. Study subjects were identified for inclusion through the RDW. For eligible patients, the following data were collected: demographics, SSTI ICD-9 codes, CPT codes for IND, wound/tissue culture results, temperature, encounter dates, and clinic location.

### Data validation and supplemental data collection

A chart review-based validation was performed to confirm SSTI diagnoses (the classification of SSTIs based on detailed chart review is hereafter referred to as the “gold standard”). During their assessment, reviewers determined the appropriate diagnosis and associated ICD-9 diagnosis code for each encounter using physician notes to ascertain the existence of an SSTI and specific type of infection by reviewing clinician notes pertaining to the body site(s) of infection, presence of erythema, purulence, spontaneous drainage, crusting, discoloration, identification and number of nodules/papules, and follicular involvement. Patient characteristics and past medical history were also considered. In cases where incision and drainage was performed or spontaneous drainage was noted, the infection was considered to be purulent. Reviewers also used provider notes and other encounter data to determine whether the encounter was an initial or follow-up visit for the SSTI. If minimal documentation was present, the provider’s diagnosis (i.e., SSTI based on ICD-9 code) was considered valid. To reduce inter-rater variability, all reviewers received study-specific training and standardized documentation for assessing SSTI diagnoses were developed based upon clinical infectious disease texts.[11-14] Reviewers extracted data into a Microsoft Access form (2007, Microsoft Corporation) to standardize the collection of data and the information used to confirm the diagnosis. Medical records were reviewed independently by at least two members of the study team to further improve reliability. If reviewers disagreed, a third reviewer served as the tie-breaker. Data extracted during the detailed chart review were stored in a Microsoft Access database.

### Data analysis

We created two algorithms for the identification of any SSTI and one algorithm for the identification of only cellulitis/abscess using EHR data. For each algorithm, the PPV was calculated using the chart review-based SSTI diagnosis as the gold standard. The three different algorithms were as follows: (1) presence of any SSTI ICD-9 [035, 680–684, 686.x, 704.8] or IND CPT code [10060/1, 10080/1, 10120/1, 10140, 10160, 10180], or a positive wound culture; (2) presence of any SSTI ICD-9 code; (3) presence of SSTI ICD-9 codes for cellulitis/abscess [681.00, 681.0, 681.1, 681.10, 681.9, 682.x]. For cellulitis/abscess diagnoses, an additional PPV was calculated where true positives were both correctly coded and body-site specific (e.g., a leg abscess confirmed by chart review was correctly identified with the ICD-9 code specifying cellulitis and abscess of leg – 682.6). Table 1 describes the different SSTI identification algorithms for which PPVs were calculated and the criteria used to identify true positives. All PPVs were calculated using both the total study sample and initial (i.e., not follow-up) encounters only. Descriptive statistics were calculated to describe demographic information such as age and gender, initial visit status, and encounter department. All data were analyzed with SAS (version 9.2, SAS Corporation, Cary NC).

**Table 1:** Description of skin and soft tissue infection identification algorithms evaluated

**Test criteria**	**Definition of true positive**
Any of the following criteria:	Any SSTI present
SSTI ICD-9 code	
Incision and drainage CPT code	
Positive wound culture	
SSTI ICD-9 code	Any SSTI present
Cellulitis/Abscess ICD-9 code	Cellulitis and/or abscess present
Cellulitis/Abscess ICD-9 code, body site specific	Cellulitis and/or abscess present at body site indicated by ICD-9 code

**Note:** SSTI, skin and soft tissue infection; ICD-9, International Classification of Diseases, Ninth Revision; CPT, current procedural terminology.

## Results

Through the electronic data warehouse, 737 of 46,045 encounters were identified that met all inclusion criteria. After chart review, 6 were excluded due to missing data in the EHR. Thus 731 encounters were included in the final analysis dataset. Of these, 54.4% were for female patients and the mean patient age was 39.1 years (standard deviation, ±23.7 years); 70.3% of the encounters were the initial visit for diagnosis and treatment of the SSTI (Table 2). Family medicine had the largest proportion of visits (61.4%) of the three outpatient departments and 13.2% of those visits were in patients under age 18. Cellulitis/abscess was the most common SSTI (68.3%) identified through chart review, followed by folliculitis (8.9%) and impetigo (8.9%), carbuncle/furuncle (6.0%), and other SSTIs (7.8%). Of the 100 wound cultures performed among the 617 SSTIs confirmed through chart review, 66 (66.0%) were positive cultures. *S. aureus* was the most frequently isolated pathogen. Among initial encounters for SSTIs, antibiotic treatment alone was prescribed in 68.4% of visits, IND alone was performed in 3.9%, IND and antibiotics were given in 11.9%, and neither IND nor antibiotic treatment was given in 15.8% of encounters. Trimethoprim/sulfamethoxazole (28.0%), cephalexin (19.2%) and mupirocin (16.2) were the most commonly prescribed antibiotics.

**Table 2:** Patient demographics and encounter characteristics

	**Family medicine**	**Internal medicine**	**Pediatrics**	**Overall**
	**(n=449)**	**(n=163)**	**(n=119)**	**(n=731)**
Age, mean years ± SD	42.9 ± 21.0	53.1 ± 15.0	5.5 ± 5.4	39.1 ± 23.7
Female sex	257 (57.2)	88 (54.0)	53 (44.5)	398 (54.4)
Race				
White	413 (92.0)	145 (89.0)	93 (78.2)	651 (89.1)
Black	11 (2.4)	11 (6.7)	7 (5.9)	29 (4.0)
Asian/Pacific Islander	13 (2.9)	2 (1.2)	11 (9.2)	26 (3.6)
Other	5 (1.1)	4 (2.5)	6 (5.0)	15 (2.1)
Unknown/not reported	7 (1.6)	1 (0.6)	2 (1.7)	10 (1.4)
Hispanic ethnicity	8 (1.8)	1 (0.6)	26 (21.8)	35 (4.8)
Initial encounter for SSTI	290 (64.6)	115 (70.6)	109 (91.6)	514 (70.3)
Encounter month				
January	116 (25.8)	27 (16.6)	27 (22.7)	170 (23.3)
April	98 (21.8)	40 (24.5)	42 (35.3)	180 (24.6)
July	116 (25.8)	48 (29.4)	30 (25.2)	194 (26.5)
October	119 (26.5)	48 (29.4)	20 (16.8)	187 (25.6)
ICD-9 code for SSTI	449 (100.0)	163 (100.0)	119 (100.0)	731 (100.0)
CPT code for IND	61 (13.6)	11 (6.7)	4 (3.4)	76 (10.4)
Positive wound culture	62 (13.8)	16 (9.8)	25 (21.0)	103 (14.1)
SSTI Identification Criteria				
ICD-9 or CPT code or positive culture	449 (100.0)	163 (100.0)	119 (100.0)	731 (100.0)
ICD-9 SSTI code only	412 (91.8)	147 (90.2)	110 (92.4)	669 (91.5)
Cellulitis/Abscess ICD-9 code	305 (67.9)	101 (62.0)	42 (35.3)	448 (61.3)
Cellulitis/Abscess ICD-9 code, body site specific	267 (59.5)	86 (52.8)	35 (29.4)	388 (53.1)

Note: Data are no. (%), unless otherwise indicated. The “Other” race category includes Native Americans, Alaskan Natives, and individuals reporting multiple races.

SD, standard deviation, SSTI, Skin and soft tissue infection,ICD-9, International Classification of Diseases, 9^th^ Revision, CPT, Current Procedure Terminology, IND, Incision and drainage.

“True” SSTIs were confirmed in 617 of the encounters, a prevalence of 1.3%. Table 3 presents the positive predictive value for each of the SSTI identification algorithms calculated among all encounters and among only initial encounters for the SSTI. The highest PPV was for detecting cellulitis/abscess based on ICD-9 codes at (PPV = 91.5%; 95% CI: 88.9-94.1%), and the lowest was for the identification cellulitis/abscess specific to body site (PPV = 52.6%; 95% CI: 47.6-57.6%). PPVs were lower overall when the SSTI identification algorithm was restricted to initial visits.

**Table 3:** Positive predictive values for each skin and soft tissue infection identification algorithm

	**Initial visits**		**All visits**	
**SSTI identification criteria**	**True positives/encounters**	**PPV (95% CI)**	**True positives/encounters**	**PPV (95% CI)**
ICD-9 or CPT code or positive culture	413/514	80.4 (76.9-83.8)	617/731	84.4 (81.8-87.0)
ICD-9 SSTI code only	404/455	88.8 (85.9-91.7)	607/669	90.7 (88.5-92.9)
Cellulitis/Abscess ICD-9 code	228/254	89.8 (086.0-93.5)	410/448	91.5 (88.9-94.1)
Cellulitis/Abscess ICD-9 code, body site specific	116/214	54.2 (47.5-60.9)	204/388	52.6 (47.6-57.6)

Note: SSTI, skin and soft tissue infection; ICD-9, International Classification of Diseases, Ninth Revision; CPT, current procedural terminology; CI, confidence interval.

## Discussion

This study demonstrated that ICD-9 codes may be used to identify SSTIs in primary care outpatient settings with a high PPV. While we had hypothesized that broadening our SSTI identification algorithm to utilize additional clinical data (i.e., wound cultures and procedure codes) would improve the performance of our algorithm, of the 61 additional encounters included with these expanded criteria, only 10 were true SSTIs. Consequently, inclusion of these data resulted in a reduction in PPV compared to an algorithm based only on ICD-9 codes.

Our study is the first to assess the PPV for EHR-based algorithms for the identification of SSTIs in a primary care outpatient setting. An earlier study by Tracy et al. evaluated the PPV of clinical cultures positive for *S. aureus* for the identification of non-invasive *S. aureus* infections in a Veterans Affairs patient population. While this study noted a high PPV for SSTIs (PPV = 95%; 95% CI: 86-98%), it is important to note this approach does not detect uncultured infections [15]. In our primary care patient sample, only 36 (5%) of patients had a positive culture for *S. aureus*. Thus, depending on the research question, an ICD-9 based method of case identification may more appropriately capture cases of SSTIs for study.

The range of PPVs observed across the different algorithms illustrates the importance of validation. In this study, we measured high PPVs using ICD-9 codes to detect SSTIs which means in turn that few patients are likely to be misclassified as an SSTI case. Still, because our study did not include patients without SSTI ICD-9 codes, we were unable to measure sensitivity. As a result, the false negative rate (i.e., misclassifying true SSTIs as non-cases) by applying an algorithm based on ICD-9 codes remains unknown. It should also be noted that, as PPV varies with prevalence, this validation study may not be generalizable to other patient populations. Also, in this patient population, providers coded infections themselves within the EpicCare EHR system and thus PPV may vary in settings where providers do not perform the coding or in which the method of coding (e.g., the EHR system) varies. Our study also did not address more complicated/severe infections such as diabetic foot infections, infected pressure ulcers, or surgical site infections. Finally, given that this was a retrospective study, the gold standard was based upon chart review and thus limited by the level of detail in the provider’s notes.

Of note, our study revealed that after evaluation of treatment patterns for true SSTIs, 33.0% of encounters (15.8% of initial encounters) received no antibiotic treatment or IND. While smaller, less severe SSTIs may not require medical intervention, the relatively large proportion of untreated patients may reflect the inclusion of follow-up visits in this study.

## Conclusion

This study demonstrates that algorithms which use ICD-9 codes to detect SSTIs can achieve a high PPV in ambulatory primary care settings. While the number of SSTI cases that would not be detected by this approach was unmeasured, the ICD-9 based SSTI identification method would likely capture those patients with a single diagnosis for their visit. Thus, these diagnosis codes may be useful in facilitating internal process improvement and quality initiatives as well as future studies exploring both the epidemiology and outcomes associated with SSTIs.

## Abbreviations

CI: Confidence intervals; CPT: Current procedural terminology; EHR: Electronic health record; ICD-9: International classification of diseases- Ninth Revision; IND: Incision and drainage; OHSU: Oregon Health & Science University; PPV: Positive predictive value; RDW: Research data warehouse; SSTI: Skin and soft tissue infections.

## Competing interests

R.K. is a member of the speakers’ bureau for Cubist Pharmaceuticals. All other authors have no other potential competing interests to report.

## Authors’ contributions

PL, JMT, DTB, and JCM contributed to the study’s inception and design. PL, MRE, and JCM drafted the manuscript and shared in data collection, interpretation, and analysis. MRE provided statistical analysis. RK, RVT, and IM significantly contributed to data collection and interpretation. All authors participated in critical revisions as well as read and approved the final manuscript.

## Pre-publication history

The pre-publication history for this paper can be accessed here:

http://www.biomedcentral.com/1471-2334/13/171/prepub
